# Genomic Changes within a Subset of IncI2 Plasmids Associated with Dissemination of *mcr-1* Genes and Other Important Antimicrobial Resistance Determinants

**DOI:** 10.3390/antibiotics11020181

**Published:** 2022-01-29

**Authors:** Nicole Ricker, Gabhan Chalmers, Elli Whalen, Heather K. Allen, Richard J. Meinersmann

**Affiliations:** 1 Food Safety and Enteric Pathogens Research Unit, ARS-USDA National Animal Disease Center, Ames, IA 50010, USA; 2 Agricultural and Biosystems Engineering, Iowa State University, Ames, IA 50011, USA; 3 Department of Pathobiology, University of Guelph, Guelph, ON N1G 2W1, Canada; gchalmer@uoguelph.ca; 4 National Poultry Research Center, USDA Agricultural Research Service, PMSP-RU, Athens, GA 30605, USA

**Keywords:** *mcr-1*, IncI2, antibiotic resistance, insertion sequences, beta-lactamase, plasmids

## Abstract

IncI2 plasmids appear to have only recently become associated with resistance genes; however, their tendency to carry resistance to the antibiotics of last resort and their widespread distribution increase their relative importance. In this study, we describe lineages within this plasmid family that have an increased likelihood of acquisition of antimicrobial resistance genes. Globally distributed *mcr-1*-carrying IncI2 plasmids were found to cluster with other IncI2 plasmids carrying extended-spectrum beta-lactamase genes, and separately from the non-resistant IncI2 plasmids. In addition, insertion sequence (IS) elements with no direct association with the acquired resistance genes also clustered with the resistance plasmids in the phylogenetic tree. In recognition of the biased sequencing of resistant plasmids globally, the analysis was also performed on resistant and non-resistant IncI2 plasmids sequenced in the USA through government surveillance efforts that do not rely on antibiotic selection. This analysis confirmed a distinct clustering associated with both resistance and mobile elements and identified possible genomic changes in core genes that correlate with increased acquisition of foreign DNA. This work highlights a potential genetic mechanism for increased uptake of foreign DNA within this prevalent family of plasmids.

## 1. Introduction

The family of IncI plasmids have been identified as the most frequent assigned incompatibility type among currently sequenced plasmids [1]. IncI2 plasmids in particular have recently garnered increased attention due to their association with extended-spectrum beta-lactamase (ESBL) genes [2,3] and the first mobile colistin resistance gene (*mcr-1*) [4]. The emergence of plasmid-mediated colistin resistance has highlighted the potentially devastating consequences of the ongoing dissemination of novel antibiotic resistance genes without detection [5,6]. Although colistin resistance had previously been documented in several pathogens (for a review, see [7]), the known mechanisms were chromosomally associated and therefore unlikely to become mobilized via plasmids among different bacterial lineages. However, the discovery by Liu et al. [4] of a novel resistance mechanism that was conferred by a single gene on a conjugative plasmid changed this paradigm and has led to an international effort to determine how widely this extra-chromosomal type of resistance has been circulating, and the factors that led to its development. Retrospective sampling indicates that *mcr-1*-containing plasmids have been present in food-producing animals for several decades in China, where it has been shown to be increasing in prevalence in recent years [8]. As has already been described by several researchers [9,10,11], IS*Apl1* mobilization of the *mcr-1* cassette through the action of Tn*6330* has facilitated its dissemination among different plasmid lineages, and stabilization has occurred through the loss of the IS*Apl1* in individual plasmids [12]. The mechanism by which the *mcr-1* cassette has been acquired and stabilized may differ among the plasmid families, as demonstrated by patterns in the variability in the flanking sequences for specific plasmid families.

In the USA, detection of *mcr-1*-containing plasmids has been rare. Through the USDA Food Safety Inspection Service (FSIS) program, *Enterobacteriaceae* isolates obtained from turkey, swine, chicken and cattle samples acquired at slaughter were analyzed for the presence of the *mcr-1* gene. From the screened samples, two isolates (both from swine) were found to be carrying colistin-resistant plasmids [13]. Both strains were fully sequenced [14,15] and *mcr-1* genes were found to be carried on IncI2 plasmids with high similarity to the initial *mcr-1* plasmid identified in China [4]. In a subsequent multi-locus sequence (MLST) study of a relatively small population of IncI2 plasmids (*n* = 81) [16] it was shown that multiple mechanisms were involved in remodeling this group of plasmids. These included transposons, homologous recombination, insertion/deletions and programmed re-assortment (specifically for *pilV*). In the current study herein, we extend the exploration to include single nucleotide polymorphism (SNP) analyses to see if this increases the resolution for characterization of these changes. In this paper, we evaluated the genetic diversity in globally distributed IncI2 plasmids to examine the emergence of IncI2 plasmids carrying antimicrobial resistance (AMR) genes. Recognizing the inherent bias in these sequenced isolates (due to increased global surveillance of AMR plasmids), we performed a separate analysis on the subset of IncI2 plasmids that have been sequenced in the USA to better understand the genetic changes that may have facilitated acquisition of antibiotic resistance genes within this plasmid family.

## 2. Results

### 2.1. Global IncI2 Diversity

**Figure 1:**
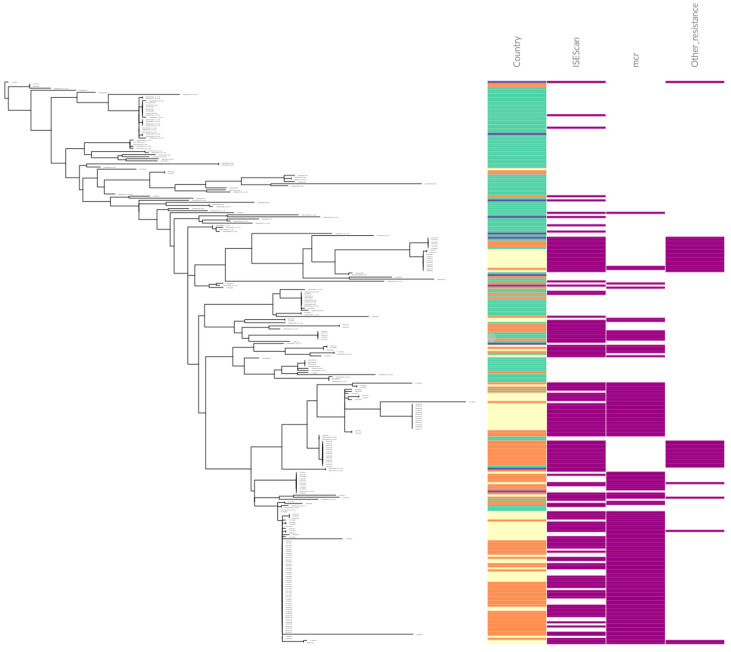
Phylogenetic tree of global IncI2 plasmids based on alignment of core genes (see methods). Country designations are USA (teal), China (beige), UK (blue) and other (orange). The remaining columns are presence or absence of IS elements, *mcr* genes, or other resistance genes, respectively. Supplemental Table S1 contains all associated metadata in the order presented in this figure.

**Figure 2:**
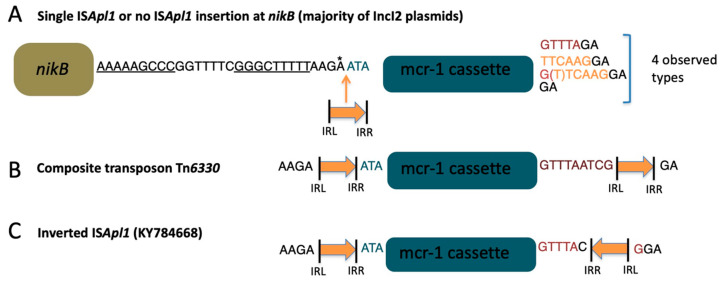
Sequences surrounding the *mcr-1* cassette in the IncI2 plasmids characterized in this study. The mcr-1 cassette includes the *mcr-1* gene and the PAP-2 hypothetical protein, as well as flanking sequences that are conserved among all the plasmids in this study. IRL and IRR refer to the left and right inverted repeats respectively for IS*Apl1* (orange arrow). (**A**) Palindromic sequence (7 bp central core flanked by 9 bp inverted repeats underlined in figure) found downstream of the *nikB* gene and adjacent to most *mcr-1* insertions in IncI2 plasmids. The final ‘A’ in this sequence (denoted with a star) is present in cases with IS*Apl1*, and absent when IS*Apl1* is absent. The sequences in blue appear to have inserted with the *mcr-1* cassette. The upstream sequence in black is present in the IncI2 plasmids without *mcr-1*. Downstream of the mcr-1 cassette shows greater variability. The sequences in red correspond with the original sequence from the composite transposon (shown in B), sequences in orange are consistent with the end of the IRR in the composite transposon and may represent a remnant after loss of the downstream IS*Apl1*. (**B**) The composite transposon Tn*6330* consists of the *mcr-1* cassette flanked by two copies of IS*Apl1*. The sequences highlighted in red occur between the end of the *mcr-1* cassette and the inverted repeat of the IS*Apl1* element located downstream of the cassette (relative to *mcr-1* transcription). (**C**) Only one composite transposon has an inverted IS*Apl1* at the downstream end, and this inversion appears to have centered around the ‘GTTTA’ sequence between the *mcr-1* cassette and the inverted repeat for IS*Apl1*.

To investigate the relationship between globally distributed IncI2 plasmids, a total of 261 plasmids were extracted from public databases including NCBI’s GenBank database and USA government surveillance efforts (see methods and Supplemental Table S1). Within the IncI2 plasmids carrying an identified *mcr-1* gene (*n* = 121), the vast majority were isolated from *E. coli* (72%) or *Shigella* (18%) followed by *Salmonella* (7%) with the remaining 3% spread between diverse strains (Supplemental Table S1). The core genome phylogenetic tree (see methods) was rooted by the original IncI2 plasmid described (AP002527) and indicated that the majority of *mcr-1*-containing plasmids formed a highly related clade (Figure 1), as previously described [16]. The remainder of the *mcr-1*-containing plasmids, and IncI2 plasmids carrying other resistance genes, also occur in the portions of the tree most distant from the chosen root; however, whether the clustering of these plasmids was artificially influenced by biased sequencing of resistant plasmids could not be determined. For this reason, we selected a subset of IncI2 plasmids for further analysis (see next section). Consistent with other reports, the *mcr-1* insertion has occurred almost exclusively in a single location (downstream of the *nikB* gene) suggesting either that these plasmids have descended from a common ancestor or that there is some form of targeted insertion for this location. Examination of the adjacent sequence revealed a conserved palindrome (Figure 2A) immediately downstream of *nikB* that could play a role in a targeted insertion in this location [16]. The majority of IncI2 plasmids carrying *mcr-1* have either a single copy of IS*Apl*1, or no copy, and variability in the flanking sequences illustrates multiple losses or rearrangements of the original composite transposon (Figure 2B,C). Consistent with the interpretation of a targeted insertion, analysis of an IncI2 plasmid carrying *mcr-7* (MG267386) revealed that this resistance gene is also inserted directly adjacent to the palindrome downstream of the *nikB* gene. Although there is limited information available on the mechanisms for this mobilization [17], the lack of sequence similarity between *mcr-1* and *mcr-7* could indicate independent acquisition events in this location.

Within the phylogenetic tree, the IncI2 plasmids did not segregate according to collection date, country of origin, or isolation source of the strain. However, some trends emerged, such as the observation that the majority of IncI2 plasmids have been isolated from either China or the USA (Figure 1) and that almost half of the IncI2 plasmids containing the *mcr-1* gene were isolated in China (54/121, 45%). Notably, all of the isolates sequenced from China (with one exception—CP010220 isolated from mouse feces) were carrying some form of antibiotic resistance gene, presumably due to sample selection bias through extensive research into antibiotic resistance prevalence there (for a review, see [18]). Conversely, most strains containing IncI2 plasmids lacking the *mcr-1* gene (100/140, 71%) were isolated in the USA through government surveillance programs. These programs perform whole-genome sequencing projects of potential pathogenic importance, and those containing IncI2 plasmids were therefore not specifically chosen based on phenotypic resistance.

In examining the collection of IncI2 plasmids that were not carrying resistance genes, we were intrigued by the finding that a substantial proportion (99/121, 82%) also did not carry any IS elements as determined by our analysis (Table 1). Although this is an expected finding for the IS elements associated with the resistance genes observed (for example IS*30*, IS*1182* and IS*1380*), there were additional IS elements (e.g., IS*200/605*, IS*1*) that were over-represented in IncI2 plasmids carrying resistance genes.

In addition to our phylogenetic analysis based on core genes, MOB-suite was used to perform whole-sequence-based assignment to MOB clustering groups. This revealed 6 sub-clusters (secondary nodes AI345-AI350) within the IncI2 collection and confirmed that the *mcr-1* plasmids were a closely related cluster as they were primarily associated with the sub-node designated AI350 (92% of *mcr* plasmids were within this node). IS element distribution was also found to differ according to the 6 designated nodes (Supplemental Table S2). AI345 and AI346 had less than 10% of their plasmids associated with IS elements (0/16 and 3/38, respectively) and 34% (17/49) of plasmids in node AI347 were associated with IS elements (primarily IS*200/605* alone or in combination with IS*30*-*mcr-1*). AI348 had a higher percentage of plasmids carrying IS elements (65%, 17/26) but notably all the plasmids carrying IS elements in this node were associated with beta-lactamase genes. Likewise, although 75% of the representatives for AI349 were carrying IS elements, this is not considered representative as there were only 8 plasmids assigned to this group. Finally, 70% of the plasmids associated with node AI350 (86/123) were found to carry IS elements, although the high abundance of *mcr-1* carrying plasmids in this group biases this result. Notably, of the plasmids in node AI350 carrying *mcr-1*, 27 of these did not carry any IS elements consistent with previous reports of the IS*Apl*1 element being lost subsequent to *mcr-1* insertion.

### 2.2. Comparison of IncI2 Plasmids from USA Surveillance

The plasmids sequenced through USA surveillance efforts represented a greater diversity of plasmids lacking resistance genes (97/106) and therefore provided an opportunity to test the hypothesis that acquisition of mobile elements (including but not limited to those carrying antibiotic resistance genes) was related to genetic differences in the core genes of the IncI2 plasmids. This collection was also balanced in terms of bacterial hosts, with 56% of the plasmids isolated from *Salmonella* (59/106) and 42% from *E. coli* (44/106). There were also two plasmids isolated from *Klebsiella* and a single plasmid from *Shigella* in this collection.

**Figure 3:**
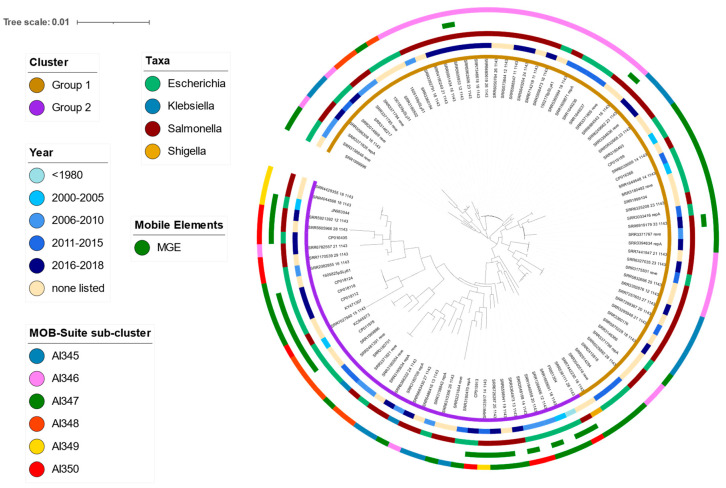
Phylogenetic analysis of the core gene SNP analysis for the USA subset of IncI2 plasmids. Rings (starting from inner to outer) correspond with: (1) designation of Group 1 or 2 based on core gene phylogeny, (2) year of isolation, (3) taxa, (4) presence or absence of mobile genetic elements and, (5) sub-cluster assigned using MOB-suite. All colors are indicated in the legends. Figure created using iTOL [19]. Analysis using MOB-suite confirmed that representatives from all 6 sub-nodes identified in the full dataset were also present in the smaller USA dataset. As the *mcr-1* plasmids were primarily associated with AI350, it was not surprising that the USA plasmids were less prevalently found in the AI350 node (*n* = 10) in this dataset and were instead more commonly assigned to the AI345-347 nodes (*n* = 15, 33 and 35 respectively). Notably, IS elements continued to be absent from plasmids assigned to node AI345 and AI346, consistent with the results of the larger dataset. The plasmids carrying *mcr-1* were divided between AI350 and AI347, suggesting separate acquisition/introduction events for *mcr-1* plasmids in the USA. An examination of the association between the core SNP phylogeny and the assigned MOB nodes indicated that changes within the core genes could be occurring broadly within IncI2 plasmids, as opposed to being isolated to a closely related node. Similar to the pattern observed for IS elements, nodes AI348-350 were primarily associated with Group 2 (67%, 100% and 100%, respectively). Of the plasmids assigned to AI347, 46% were associated with Group 2, which is slightly higher than the observed association of IS elements with this node in the full dataset (35%). Despite a large number of representatives, only 6% of plasmids in node AI346 (2/33) were associated with Group 2. Interestingly, 27% of AI345 plasmids (4/15) were associated with Group 2, although there were no plasmids from this node found to carry any IS elements in either dataset.

The pangenome analysis of USA IncI2 plasmids illustrated that they had similar overall diversity to the full IncI2 collection analyzed previously. There were 29 core genes identified in the USA dataset (Supplemental Table S3), and a total of 260 unique genes identified (compared to 27 core and 264 total genes for the full IncI2 collection). Most of the plasmids in the USA collection did not contain any IS elements (Table 2) and all except three plasmids carrying IS elements clustered together based on SNP phylogeny of core genes and will be referred to as Group 2 (Figure 3). Linkage analysis confirmed that the clade of plasmids in Group 2 shows greater linkages among alleles, particularly in relation to genes involved in conjugation and pilus formation (Supplemental Table S4). Although many of the plasmid submissions did not include isolation dates, it should be noted that the oldest plasmid in the collection (FR851304, isolation date 1979) is found within Group 2, suggesting that this branch represents a separate trajectory of plasmids as opposed to a recent divergence. Among the accessory genes strongly correlated with this clade were the molecular chaperone *dnaJ* as well as a primosomal protein *dnaT*. Of note, the addiction gene *relE* was also found solely within this clade of plasmids (with one exception—SRR3158846), which may be important for understanding the host range and stability of this subset of plasmids.

Analysis using MOB-suite confirmed that representatives from all 6 sub-nodes identified in the full dataset were also present in the smaller USA dataset. As the *mcr-1* plasmids were primarily associated with AI350, it was not surprising that the USA plasmids were less prevalently found in the AI350 node (*n* = 10) in this dataset and were instead more commonly assigned to the AI345-347 nodes (*n* = 15, 33 and 35 respectively). Notably, IS elements continued to be absent from plasmids assigned to node AI345 and AI346, consistent with the results of the larger dataset. The plasmids carrying *mcr-1* were divided between AI350 and AI347, suggesting separate acquisition/introduction events for *mcr-1* plasmids in the USA. An examination of the association between the core SNP phylogeny and the assigned MOB nodes indicated that changes within the core genes could be occurring broadly within IncI2 plasmids, as opposed to being isolated to a closely related node. Similar to the pattern observed for IS elements, nodes AI348-350 were primarily associated with Group 2 (67%, 100% and 100%, respectively). Of the plasmids assigned to AI347, 46% were associated with Group 2, which is slightly higher than the observed association of IS elements with this node in the full dataset (35%). Despite a large number of representatives, only 6% of plasmids in node AI346 (2/33) were associated with Group 2. Interestingly, 27% of AI345 plasmids (4/15) were associated with Group 2, although there were no plasmids from this node found to carry any IS elements in either dataset.

## 3. Discussion

The first discovery of *mcr-1* [4] set off an intensive search for the gene in many parts of the world. It was found fairly frequently in Southeast Asia and less often in the rest of the world but widely scattered in all the continents save Antartica [20]. Most of the isolations came from farm animals, notably pigs. At the time of the first isolation the use of colistin was not regulated in much of Southeast Asia and was commonly used for promoting growth [20] and for prophylaxis for gastrointestinal disease during weaning [21]. It is apparent that the use of colistin created selective pressure favoring bacteria carrying *mcr-1*. The gene was most commonly found on IncI2 plasmids [22].

IncI2 plasmids appear to have only recently become associated with resistance genes; however, their tendency to carry resistance to the antibiotics of last resort and their widespread distribution increase their relative importance. Both the β-lactamase- and *mcr-1*-containing plasmids clustered together in the IncI2 phylogenetic analysis, and this cluster of plasmids was also more likely to be carrying mobile genetic elements (MGEs), including MGEs that were not directly related to acquisition of the resistance genes. Although the clustering may have resulted from strong bias in the representative isolates that have been sequenced, the clustering of plasmids containing increased accessory genes was also evident when examining the collection of IncI2 plasmids sequenced through multiple surveillance networks within the USA. Our results illustrate that the IncI2 plasmids that were part of the resistance cluster had a greater diversity of accessory genes and shared several mobile elements that were not specifically associated with the resistance genes that they carried, and that were missing from the other clade of IncI2 plasmids. Identifying the source of these shared mobile elements (other co-resident plasmids vs. direct acquisition through DNA uptake) could aid in identifying potential routes of resistance gene acquisition in this family of plasmids.

In conclusion, we have demonstrated increased prevalence of IS elements and AMR genes within a subset of IncI2 plasmids and that *mcr* gene acquisition may be associated with a short palindrome sequence directly downstream of the *nikB* gene. In addition, our core gene SNP analysis identified strong linkages of allelic variants that could indicate evolution towards increased acquisition of MGEs within the IncI2 family of plasmids. Future work aimed at determining whether the acquisition of certain genes or specific mutations within core genes has facilitated increased gene flexibility within this subset of plasmids, would be pertinent. Our work suggests that mutational changes in conjugation-related genes, or specific gene acquisitions such as *relE*, could influence host range or plasmid stability and therefore impact the evolutionary path of this family of plasmids. As IncI2 plasmids are among the most prevalent *mcr-1*-carrying plasmids globally and are also found in the greatest diversity of bacterial hosts and geographic locations [23], understanding the selective pressures and evolution of this subset of IncI2 plasmids could potentially direct future research and identify key bacterial hosts, isolation sources, or co-occurring plasmids that have influenced the acquisition of mobile elements in these highly disseminated plasmids.

## 4. Materials and Methods

### 4.1. Phylogenetic Reconstruction of IncI2 Plasmids

A total of 261 plasmids (listed in Table S1) were collected from public databases and government surveillance programs in the USA. Genes were annotated using Prokka v1.14.6 [24] and a general time reversible phylogenetic tree was made in FastTree v2.1.11 [25] using the core genome alignment produced by Roary v3.13.0 [26] with the original described IncI2 plasmid (AP002527) designated as the root. Visualizations of constructed trees and pangenome content were created using Phandango [27] and iTOL [19]. MOB-suite v3.0.3 was used to reconstruct plasmid content from each assembly. MOB-recon was used to analyze plasmid sequences, which includes MOB_typer to perform relaxase and replicon typing of plasmids, as well as generate MOB-cluster codes and host range information.

### 4.2. Analysis of USA Plasmids

We undertook a separate phylogenetic analysis of USA plasmids due to the extent of isolates available through government surveillance programs that did not specifically rely on antibiotic selection for isolate acquisition. A total of 106 plasmids with the source designations of “US”, “USA”, “Pulsenet”, “GenomeTrakr”, “FSIS” and “NARST” were analyzed separately as the USA cohort. Alignment of the USA cohort was performed using the MAFFT v7.308 [28] in Geneious, and the same phylogenetic analysis as described above was performed using the Compute Canada Graham cluster. To determine linkage between loci among the plasmids, the linkage allele table described by Meinersmann [16] was extended to include the new plasmids of interest. The table was imported into Arlequin v3.5.2.2 [29]. The exact test of linkage disequilibrium [30] and a locus-by-locus AMOVA with population specific Fst’s [31] were calculated.

## Figures and Tables

**Table 1:** Distribution of IS elements in global collection of IncI2 plasmids correlated with carriage of resistance genes.

ISEScan	No AMR	*mcr-1*	*mcr-7.1_1*	*aadA, dfrA*	*bla*_CMY-2_1_	*bla*_CTX-M-132_1_	*bla*_CTX-M-15_1_	*bla*_CTX-M-199_1_	*bla*_CTX-M-55_1_	*bla*_CTX-M-64_1_	*bla_KPC_*_-3_, *aac(6’)-Ib*, *aadA*, *bla*_OXA-9_, *bla*_TEM-1_	*bla*_KPC-3_, *bla*_TEM-1_
No IS found	99	32	-	-	-	-	-	-	-	-	-	-
IS*1*	-	2	-	-	-	-	-	-	-	-	-	-
IS*1*, IS*200*/IS*605*	-	13	-	-	-	-	-	-	-	-	-	-
IS*1*, IS*30*, IS*91*	-	2	-	-	-	-	-	-	-	-	-	-
IS*1*, IS*5*	-	1	-	-	-	-	-	-	-	-	-	-
IS*110*, IS*3*, IS*30*	-	1	-	-	-	-	-	-	-	-	-	-
IS*1182*, IS*21*	-	-	-	-	-	-	-	-	-	-	3	2
IS*1182*, IS*21*, IS*91*	-	-	-	-	-	-	-	-	-	-	1	-
IS*1380*	-	3	1	-	13	2	-	1	6	3	-	-
IS*1380*, IS*3*, IS*30*	-	1	-	-	-	-	-	-	1	-	-	-
IS*1380*, IS*3*, IS*91*	-	-	-	-	-	-	1	-	-	-	-	-
IS*1380*, IS*30*	-	2	-	-	-	-	-	-	2	-	-	-
IS*200*/IS*605*	15	11	-	-	-	-	-	-	-	-	-	-
IS*200*/IS*605*, IS*3*	-	1	-	-	-	-	-	-	-	-	-	-
IS*200*/IS*605*, IS*30*	1	9	-	-	-	-	-	-	-	-	-	-
IS*200*/IS*605*, IS*91*	1	1	-	-	-	-	-	-	-	-	-	-
IS*3*	3	3	-	1	-	-	-	-	-	-	-	-
IS*3*, IS*30*	-	1	-	-	-	-	-	-	-	-	-	-
IS*3*, IS*91*	-	1	-	-	-	-	-	-	-	-	-	-
IS*30*	1	20	-	-	-	-	-	-	-	-	-	-
IS*30*, IS*5*	-	1	-	-	-	-	-	-	-	-	-	-
IS*30*, IS*630*	-	1	-	-	-	-	-	-	-	-	-	-
IS*30*, IS*66*	-	2	-	-	-	-	-	-	-	-	-	-
IS*30*, IS*91*	-	1	-	-	-	-	-	-	-	-	-	-
IS*4*	-	4	-	-	-	-	-	-	-	-	-	-
IS*66*	1	1	-	-	-	-	-	-	-	-	-	-
IS*91*	-	6	-	-	-	-	-	-	-	-	-	-

**Table 2:** Distribution of IS elements within USA IncI2 plasmids analyzed. Please note that IS*30*, IS*1182* and IS*1380* are the only IS elements directly associated with antibiotic resistance genes in these plasmids.

ISEScan Result	No AMR	*mcr-1.1*	*bla*_CMY-2_	*bla_KPC_*_-3_, *aac(6′)-Ib*, *aadA*, *bla*_OXA-9_, *bla*_TEM-1_
No IS found	84	-	-	-
IS*1182*, IS*21*	-	-	-	2
IS*1380*	-	-	1	-
IS*200*/IS*605*	8	2	-	-
IS*200*/IS*605*, IS*30*	1	3	-	-
IS*200*/IS*605*, IS*91*	-	1	-	-
IS*3*	2	-	-	-
IS*30*	1	-	-	-
IS*66*	1	-	-	-

## Data Availability

All sequences used in this study were acquired through publicly available databases.

## Supplementary Materials

The following supporting information can be downloaded at: https://www.mdpi.com/article/10.3390/antibiotics11020181/s1, Table S1: Metadata for global collection of IncI2 plasmids; Table S2: Mob-suite distribution for IncI2 plasmids in this study; Table S3: Core genes identified in the USA plasmids; Table S4: Linkage analysis.
