# Supplementary material for: Genomic Changes within a Subset of IncI2 Plasmids Associated with Dissemination of mcr-1 Genes and Other Important Antimicrobial Resistance Determinants
Source: Antibiotics (Basel). 2022 Jan 29;11(2):181. doi: 10.3390/antibiotics11020181 (PMC8868234; doi:10.3390/antibiotics11020181)
Supplement: Supplementary file 1 [file antibiotics-11-00181-s001.zip › antibiotics-1553129-supplementary.pdf]

antibiotics

MDPI

Supplementary Material

Supplemental Table S1: Metadata file – plasmids are listed in the same order as Figure 2.

Name	Country	Host	isolation source	Collection	Organism	ISEScan	mcr	AMR
AP002527	UK	human	urine	1972	Escherichia coli	IS3		aadA, dfrA
SRR3360194	US	human	feces	2010	Escherichia coli O103:H2			
1505159pSLy51	US	dairy			Salmonella enterica Kentucky			
SRR6782557_21_1143	US	human	feces	2018	Salmonella enterica subsp. enterica serovar Muenchen			
SRR4190249_21_1143	US		feces	2016	Salmonella enterica subsp. enterica serovar Newport			
SRR5951434_16_1143	US	human	urine	2017	Salmonella enterica subsp. enterica serovar Newport			
SRR5360994_18_1143	US		feces	2014	Salmonella enterica subsp. enterica serovar Berta			
1502178pSLy41	US	bovine			Salmonella enterica Anatum			
SRR1609871_repA_	US	human		2014	Escherichia coli			
SRR1649236	US	human		2014	Escherichia coli			
SRR1649237	US	human		2014	Escherichia coli			
SRR2102959	US	human	feces		Escherichia coli O157:H7			

SRR3371805 _reve	US	human	feces		Escherichia coli O157:H7			
SRR6984543 _16_1143	US				Salmonella enterica	IS66		
SRR5956473 _16_1143	US	swine	retail	2017	Salmonella enterica subsp. enterica serovar Muenster			
SRR5573844 _12_1143	US				Salmonella enterica			
SRR5588547 _11_1143	US	human	feces	2017	Salmonella enterica subsp. enterica serovar Enteritidis			
SRR5640204 _24_1143	US	human	peritoneal fluid	2017	Salmonella enterica subsp. enterica serovar Enteritidis			
SRR5714218 _11_1143	US				Salmonella enterica			
SRR5605933 _12_1143	US	human	feces	2017	Salmonella enterica	IS3		
SRR5962606 _23_1143	US	human	feces	2017	Salmonella enterica subsp. enterica serovar Montevideo			
SRR7346618 _19_1143	US	human	feces	2018	Salmonella enterica			
SRR1967295 _26_1143	UK	human		2014	Salmonella enterica subsp. enterica serovar Typhimurium			

SRR6050764 _26_1143	US				Salmonella enterica			
SRR5680619 _26_1143	US	human	feces	2017	Salmonella enterica subsp. enterica serovar Java			
SRR5832666 _25_1143	US				Salmonella enterica			
SRR3175501 _reve	US	human	feces	2015	Escherichia coli O157:H7			
SRR6327035 _23_1143	US	human	feces	2017	Salmonella enterica subsp. enterica serovar 4,[5],12:i:-			
SRR3392876 _12_1143	US				Salmonella enterica			
SRR7441947 _21_1143	US				Salmonella enterica			
SRR7257603 _27_1143	US	beef		2018	Salmonella enterica subsp. enterica serovar Give			
SRR7269367 _20_1143	US				Salmonella enterica			
SRR2014869 _reve	US	human		2012	Escherichia species O174:H2			
SRR6366332 _24_1143	US	human	feces	2017	Salmonella enterica subsp. enterica serovar 4,[5],12:i:-			

SRR3180709 _repA_	US	human	feces	2009	Escherichia coli O157:H7			
SRR3146217	US	human	feces	2016	Escherichia coli STEC			
SRR3180504 _reve	US	human	feces	2007	Escherichia coli O157:H7			
SRR3371821 _reve	US	human	feces	2007	Escherichia coli O157:H7			
SRR3185634 _repA_	US	human			Escherichia coli			
CP010220	China	mouse	feces	2014	Escherichia coli			
CP011291	Germany			2015	Salmonella enterica subsp diarizonae			
CP011294	Germany			2015	Salmonella enterica subsp diarizonae			
1501839pSL y31	US	bovine			Escherichia coli			
SRR3371785 _reve	US	human	feces	2010	Escherichia coli			
SRR3371794 _reve	US	human	feces	2010	Escherichia coli			
SRR3180502	US	human			Escherichia coli O157:H7			
SRR3158846 _reve	US	human	feces	2006	Escherichia coli O157:H7			
SRR3371826 _repA_	US	human	feces	2007	Escherichia coli O157:H7			
SRR1999996	US	human			Escherichia coli			

SRR3231644 _reve	US	human			Escherichia coli			
SRR5586308 _16_1143	US	human	feces	2016	Salmonella enterica subsp. enterica serovar Enteritidis			
SRR6249662 _23_1143	US	human	feces	2017	Salmonella enterica subsp. enterica serovar 4,[5],12:b:-			
CP009581	Netherlands			2014	Escherichia coli	IS200/IS60 5		
SRR3180493	US	human			Escherichia coli			
SRR1966743 _30_1143	UK	human		2014	Salmonella enterica subsp. enterica serovar Stanley	IS200/IS60 5		
SRR3158842 _repA_	US	human	feces	2007	Escherichia coli O157:NM			
SRR3394636 _reve	US	human	feces	2016	Escherichia coli O157:H7			
SRR5832668 _23_1143	US				Salmonella enterica			
SRR3180470 _repA_	US	human	feces	2013	Escherichia coli O121:H19			
SRR5364973 _13_1143	US	human	feces	2017	Salmonella enterica			
CP015913	US	swine	cecum	2016	Escherichia coli	IS200/IS60 5, IS91	mcr-1.1_1	

SRR7442230_18_1143	US				Salmonella enterica			
SRR7221396_30_1143	UK	human		2018	Salmonella enterica subsp. enterica serovar Typhimurium	IS200/IS605		
1605825pSLy61	US	dairy			Salmonella enterica Cerro			
SRR6310306_26_1143	US				Salmonella enterica			
SRR6663430_27_1143	US		feces	2018	Salmonella enterica subsp. enterica serovar Typhimurium			
SRR1266066_12_1143	US	bird	composite	2008	Salmonella enterica subsp. enterica serovar Newport	IS200/IS605		
SRR1609691_18_1143	US	turkey	retail	2005	Salmonella enterica subsp. enterica serovar Muenster			
SRR1946958_20_1143	US	turkey	retail	2007	Salmonella enterica subsp. enterica serovar Heidelberg			
SRR2648198_14_1143	US	turkey	retail	2008	Salmonella enterica subsp. enterica serovar Newport	IS200/IS605		
SRR6897879_21_1143	UK	human		2016	Salmonella enterica subsp. enterica serovar Enteritidis			

SRR2481391 _reve	US	human	feces	2015	Shigella sonnei			
CP011645	UK			2015	Klebsiella pneumoniae	IS1182, IS21		KPC-3, aac(6')-Ib, aadA, OXA-9, TEM-1
CP011978	US				Klebsiella pneumoniae	IS1182, IS21		KPC-3, aac(6')-Ib, aadA, OXA-9, TEM-1
CP021546					Klebsiella pneumoniae	IS1182, IS21		KPC-3, TEM-1
CP021754					Klebsiella pneumoniae	IS1182, IS21		KPC-3, TEM-1
CP021861					Klebsiella pneumoniae	IS1182, IS21, IS91		KPC-3, aac(6')-Ib, aadA, OXA-9, TEM-1
KC845573	US	human		2005	Klebsiella pneumoniae	IS1182, IS21		KPC-3, aac(6')-Ib, aadA, OXA-9, TEM-1
JN797501	China	dog	feces	2008	Escherichia coli	IS1380		blaCTX-M-55_1
KX827311	China				Shigella sonnei	IS1380		blaCTX-M-55_1
KF601686	China	chicken		2006	Escherichia coli	IS1380, IS3, IS91		blaCTX-M-15_1
KJ020576	China	chicken	feces	2011	Escherichia coli	IS1380		blaCTX-M-64_1

KJ460501	China	human		2013	Shigella sonnei	IS1380		blaCTX-M-55_1
KM207012	China	human		2013	Escherichia coli	IS1380		blaCTX-M-132_1
KP091735	China	chicken		2013	Escherichia coli	IS1380		blaCTX-M-64_1
KP198615	China	swine		2012	Escherichia coli	IS1380		blaCTX-M-132_1
KX034083	China	chicken	feces	2012	Escherichia coli	IS1380, IS3, IS30	mcr-1.1_1	blaCTX-M-55_1
KY012275					Escherichia coli	IS1380, IS30	mcr-1.1_1	blaCTX-M-55_1
LN623683	China	human		2008	Salmonella enterica	IS1380		blaCTX-M-55_1
SRR1999886	US	human			Escherichia coli			
SRR7191870_25_1143	UK	human		2017	Salmonella enterica subsp. enterica serovar Typhimurium			
CP019690	Hungary	human	feces	2002	Shigella sonnei			
SRR3180701	US	human	feces	2015	Escherichia coli O145:H34			
SRR7027940_15_1143	US				Salmonella enterica	IS3		
MG598816	Argentina		urine		Escherichia coli		mcr-1.1_1	

SRR5220264 _29_1143	UK	human		2016	Salmonella enterica subsp. enterica serovar Enteritidis	IS200/IS605, IS91		
MG594800	Argentina		urine		Escherichia coli		mcr-1.1_1	
SRR1649548 _14_1143	US	equine	feces	2004	Salmonella enterica subsp. enterica serovar Newport			
SRR6919179 _33_1143	US	swine	feces	2018	Salmonella enterica	IS30		
LM996894	Norway	human	feces	2010	Escherichia coli	IS3		
SRR1999104	US	human			Escherichia coli STEC			
SRR1999965								
SRR3033416 _repA_	US	human	feces	2015	Escherichia coli O121:HNT			
SRR3371767 _reve	US	human	feces	2009	Escherichia coli O121:H19			
SRR3394634 _repA_	US	human	feces	2016	Escherichia coli O157:H7			
SRR6325208 _23_1143	US				Salmonella enterica			
CP016388	US	human	blood	2013	Salmonella enterica subsp. enterica serovar Typhimurium			
SRR3180482 _reve	US	human	feces		Escherichia coli O157:H7			

CP019199	US	dairy	feces	2001	Salmonella enterica subsp. enterica serovar Muenster str. 0315			
SRR6038699_14_1143	US				Salmonella enterica			
LM995586	Norway	human	feces	2006	Escherichia coli	IS200/IS605		
KU922754	China	sewage		2016	Kluyvera ascorbata		mcr-1.1_1	
KY075654	China	human			Escherichia coli	IS4	mcr-1.1_1	
SRR6726287_25_1143	US	human	feces	2018	Salmonella enterica	IS200/IS605		
CP006264	Belgium	human		2007	Escherichia coli	IS200/IS605		
CP007134	Belgium	human		2007	Escherichia coli	IS200/IS605		
SRR5598941_19_1143	Mongolia				Salmonella enterica subsp. enterica serovar Typhimurium	IS200/IS605		
CP018106	Germany	human	clinical	2016	Escherichia coli	IS200/IS605, IS30	mcr-1.1_1	
CP018112	USA	human	clinical	2016	Escherichia coli	IS200/IS605, IS30	mcr-1.1_1	
CP018118	USA	human	clinical	2016	Escherichia coli	IS200/IS605, IS30	mcr-1.1_1	

CP018124	USA	human	clinical	2016	Escherichia coli	IS200/IS605, IS30	mcr-1.1_1	
KY471307	Canada	human			Escherichia coli	IS200/IS605	mcr-1.1_1	
FR851304	US	turkey		1979	Escherichia species	IS200/IS605		
SRR1645577_28_1143	UK	food		2012	Salmonella enterica subsp. enterica serovar Typhimurium			
KX505142	China	chicken	feces	2015	Cronobacter sakazakii	IS200/IS605, IS30	mcr-1.1_1	
MG725031		chicken	chicken		Escherichia coli	IS200/IS605	mcr-1.1_1	
MG210938	China	human	urine	2015	Escherichia coli	IS200/IS605, IS30	mcr-1.1_1	
AP018355		poultry			Escherichia coli	IS200/IS605, IS30	mcr-1.1_1	
SRR5605966_28_1143	US				Salmonella enterica	IS200/IS605, IS30	mcr-1.1_1	
CP025679	China	chicken		2014	Escherichia albertii	IS30	mcr-1.1_1	
SRR3146366	US	human	feces		Escherichia coli O118:H16			
SRR2415816	US	human	feces	2015	Escherichia coli O71:H11			
SRR2415818	US	human	feces	2015	Escherichia coli O71:H11			
SRR2914294	US	human	feces	2015	Escherichia coli O71:H11			

SRR3040516 _reve	US	human	feces	2015	Escherichia coli O71:H11			
SRR3371798 _repA_	US	human			Escherichia coli O157:H7			
SRR6025892 _28_1143	US				Salmonella enterica			
LT795506		swine	feces		Escherichia coli			
SRR5486416 _13_1143	US				Salmonella enterica			
SRR3295948 _21_1143	US	dairy		2014	Salmonella enterica subsp. enterica serovar Typhimurium			
SRR3360176	US	human			Escherichia coli STEC			
SRR5875228 _18_1143	US		raw almonds	2017	Salmonella enterica			
KY624633	Bolivia		food	2013	Citrobacter braakii	IS91	mcr-1.1_1	
KU761326	China	human		2015	Escherichia coli	IS200/IS605	mcr-1.1_1	
MG946761					Escherichia coli	IS200/IS605	mcr-1.1_1	
CP016405	US	swine	cecum	2016	Escherichia coli	IS200/IS605	mcr-1.1_1	
MK542639	Russia	bird	Black kite		Escherichia coli		mcr-1.1_1	
CP024135	China	human	clinical	2014	Escherichia coli	IS200/IS605, IS30	mcr-1.1_1	

KY075651	China	swine			Escherichia coli	IS200/IS60 5, IS3	mcr-1.1_1	
KY075662	China	swine			Escherichia coli	IS200/IS60 5	mcr-1.1_1	
MF774183	China	swine	feces		Escherichia coli	IS30, IS5	mcr-1.1_1	
KY792081					Escherichia coli		mcr-1.8_1	
MG299127	China	human		2016	Shigella sonnei	IS1, IS200/IS60	mcr-1.1_1	
MG299129	China	human		2016	Shigella sonnei	IS1, IS200/IS60	mcr-1.1_1	
MG299132	China	human		2016	Shigella sonnei	IS1, IS200/IS60	mcr-1.1_1	
MG299134	China	human		2016	Shigella sonnei	IS1, IS200/IS60	mcr-1.1_1	
MG299136	China	human		2016	Shigella sonnei	IS1, IS200/IS60	mcr-1.1_1	
MG299138	China	human		2016	Shigella sonnei	IS1, IS200/IS60	mcr-1.1_1	
MG299140	China	human		2016	Shigella sonnei	IS1, IS200/IS60	mcr-1.1_1	
MG299142	China	human		2016	Shigella sonnei	IS1, IS200/IS60	mcr-1.1_1	
MG299144	China	human		2016	Shigella sonnei	IS1, IS200/IS60	mcr-1.1_1	

MG299146	China	human		2016	Shigella sonnei	IS1, IS200/IS60	mcr-1.1_1	
MG299148	China	human		2016	Shigella sonnei	IS1, IS200/IS60	mcr-1.1_1	
MG299150	China	human		2016	Shigella sonnei	IS1, IS200/IS60	mcr-1.1_1	
MG299152	China	human		2016	Shigella sonnei	IS1, IS200/IS60	mcr-1.1_1	
KY471314	Argentina	human			Escherichia coli	IS200/IS605	mcr-1.1_1	
MF693349	Argentina	human	feces	2016	Escherichia coli	IS200/IS605	mcr-1.1_1	
KU870627	South Africa	chicken		2015	Escherichia coli	IS30	mcr-1.1_1	
SRR4044568_18_1143	US				Salmonella enterica			
SRR4429355_18_1143	US				Salmonella enterica			
CP043220	Canada	chicken	chicken	2016	Escherichia coli O80:H26	IS1380		blaCMY-2_1
JN983044	US	swine		2002	Salmonella enterica subsp enterica serovar Heidelberg	IS1380		blaCMY-2_1
SRR3048532_28_1143	UK	human			Salmonella enterica subsp. enterica serovar Typhimurium	IS200/IS605		
SRR4123917_14_1143	Tanzania	bovine		2013	Salmonella enterica subsp. enterica	IS200/IS605		

KY012274	China	human	blood	2014	Escherichia coli		mcr-1.1_1	
CP016185	Malaysia	chicken	liver	2013	Escherichia coli	IS1	mcr-1.1_1	
CP016186	Malaysia	pond water		2013	Escherichia coli		mcr-1.1_1	
CP016187	Malaysia	chicken feed		2013	Escherichia coli		mcr-1.1_1	
CP021176	Taiwan	human	urine	2016	Escherichia coli		mcr-1.1_1	
KU934209	China	chicken	intestines		Salmonella enterica	IS1380, IS30	mcr-1.1_1	blaCTX-M-55_1
KX013539	Bahrain	human	urine	2015	Escherichia coli	IS91	mcr-1.1_1	
KY120365	Taiwan	swine		2012	Salmonella enterica subsp. enterica		mcr-1.1_1	
KY795978	Australia	human	urine	2011	Escherichia coli		mcr-1.1_1	
SRR7439317_24_1143	UK	human		2017	Salmonella enterica subsp. Enterica monophasic			
KY693674	Oman	human	blood	2016	Escherichia coli	IS3	mcr-1.1_1	
KY829117	China				Escherichia coli	IS30	mcr-1.7_1	
KX013540	Bahrain	human	bed sore	2015	Escherichia coli	IS1380	mcr-1.1_1	blaCTX-M-64_1

SRR5361111 _28_1143	US				Salmonella enterica	IS200/IS605		
KY471315	Argentina	human			Escherichia coli		mcr-1.1_1	
MG598814	Argentina		urine		Escherichia coli	IS30	mcr-1.5_1	
SRR5921392 _12_1143	US	human	feces	2017	Salmonella enterica subsp. enterica serovar Enteritidis	IS200/IS605		
SRR2082855 _16_1143	US	turkey	retail	2006	Salmonella enterica subsp. enterica serovar Schwarzengrund			
SRR7170539 _29_1143	US				Salmonella enterica			
KY075661	China	swine			Escherichia coli	IS200/IS605	mcr-1.1_1	
CP024139	China	human	clinical	2014	Escherichia coli	IS200/IS605, IS30	mcr-1.1_1	
KX084393	China			2016	Escherichia coli	IS200/IS605	mcr-1.1_1	
KX254342	China				Escherichia coli	IS200/IS605	mcr-1.1_1	
AP018806	Japan	bovine	feces	2013	Escherichia coli		mcr-1.1_1	
KY075657	China	swine			Escherichia coli	IS4	mcr-1.1_1	

KY075658	China	swine			Escherichia coli	IS4	mcr-1.1_1	
CP021205	China	human	blood	2014	Escherichia coli	IS3, IS91	mcr-1.1_1	
KX592672	China	human	blood	2014	Escherichia coli	IS1	mcr-1.1_1	
KX772778	China	human		2016	Escherichia coli	IS1380	mcr-1.1_1	blaCTX-M-55_1
KX856067	China	human	feces	2013	Salmonella enterica subsp. enterica serovar Typhimurium		mcr-1.1_1	
KY363998	China	human		2011	Shigella sonnei		mcr-1.1_1	
MF774181	China	swine	feces		Escherichia coli	IS30, IS66	mcr-1.1_1	
KY784668	China	swine	feces		Shigella flexneri Y	IS110, IS3, IS30	mcr-1.1_1	
AP017614	Japan	swine		2008	Escherichia coli	IS30	mcr-1.1_1	
AP017619	Japan	bovine		2012	Escherichia coli	IS30	mcr-1.1_1	
AP017622	Japan	bovine		2013	Escherichia coli	IS30	mcr-1.1_1	
AP018110	Japan	human	urine	2016	Escherichia coli	IS30	mcr-1.5_1	
AP018412					Escherichia coli		mcr-1.1_1	

AP018812	Japan	bovine	feces	2014	Escherichia coli	IS91	mcr-1.1_1	
AP019686	Japan			2017	Escherichia coli		mcr-1.1_1	
CP021194	China	human	clinical	2012	Escherichia coli		mcr-1.1_1	
CP041113	Thailand	swine	feces	2016	Escherichia coli	IS30, IS630	mcr-1.1_1	
KP347127	China	swine		2015	Escherichia coli	IS30, IS66	mcr-1.1_1	
KU934208	China	chicken		2013	Escherichia coli		mcr-1.3_1	
KX013538	United Arab Rep	human	blood	2013	Escherichia coli	IS30	mcr-1.1_1	
KX580716					Escherichia coli	IS91	mcr-1.1_1	
KX856068	China	human	feces	2012	Salmonella enterica subsp. enterica serovar Enteritidis	IS30	mcr-1.1_1	
KY120366	Taiwan	human			Salmonella enterica subsp. enterica		mcr-1.1_1	
KY363994	China	human	feces	2012	Shigella sonnei		mcr-1.1_1	
KY363995	China	human	feces	2013	Shigella sonnei		mcr-1.1_1	
KY363996	China	human		2011	Shigella sonnei	IS30, IS91	mcr-1.1_1	

KY363997	China	human		2011	Shigella sonnei	IS1, IS30, IS91	mcr-1.1_1	
KY363999	China	human	feces	2010	Shigella sonnei	IS1, IS30, IS91	mcr-1.1_1	
KY405001	South Korea		blood		Escherichia coli	IS66	mcr-1.1_1	
KY471144					Escherichia coli	IS3	mcr-1.1_1	
KY471308	Argentina	human			Escherichia coli	IS30	mcr-1.5_1	
KY471309	Argentina	human			Escherichia coli		mcr-1.1_1	
KY471310	Argentina	human			Escherichia coli	IS30	mcr-1.5_1	
KY471311	Argentina	human			Escherichia coli	IS30	mcr-1.5_1	
KY471312	Argentina	human			Escherichia coli	IS91	mcr-1.1_1	
KY471313	Argentina	human			Escherichia coli	IS91	mcr-1.1_1	
KY795977	Australia	human	urine	2011	Escherichia coli		mcr-1.1_1	
LC469775	Japan		wastewater	2018	Escherichia coli		mcr-1.1_1	
LC473131	Japan		influent municipal wastewater		Escherichia coli		mcr-1.1_1	

LT174530	Vietnam	human	feces	2008	Shigella sonnei	IS30	mcr-1.1_1	
MF069152	China	chicken	small intestine	2012	Escherichia coli	IS30	mcr-1.1_1	
MG210940	China	human	ascites	2015	Escherichia coli	IS1, IS5	mcr-1.1_1	
MG489944	Thailand	chicken	retail		Escherichia coli	IS30	mcr-1.1_1	
MG557851	Thailand	chicken	feces		Escherichia coli	IS30	mcr-1.1_1	
MG594798	Argentina		urine		Escherichia coli	IS30	mcr-1.5_1	
MG594799	Argentina		urine		Escherichia coli		mcr-1.1_1	
MG598815	Argentina		urine		Escherichia coli		mcr-1.1_1	
MG825369		swine	retail		Escherichia coli	IS4	mcr-1.1_1	
MH271383	Argentina	dog	urine		Escherichia coli		mcr-1.1_1	
MH522410					Salmonella enterica subsp. enterica serovar Typhimurium	IS30	mcr-1.1_1	
MH522414		human			Salmonella enterica subsp. enterica serovar Typhimurium		mcr-1.1_1	

MH522416		human			Salmonella enterica subsp. enterica serovar Typhimurium		mcr-1.1_1	
MK041211	Argentina		feces		Citrobacter amalonaticus	IS30	mcr-1.5_1	
KX032519	South Africa	human	blood	2014	Escherichia coli	IS3, IS30	mcr-1.1_1	
KY075656	China	swine			Escherichia coli		mcr-1.1_1	
KY471145					Escherichia coli	IS3	mcr-1.1_1	
KY802014	China	human	feces	2015	Escherichia coli	IS1380	mcr-1.1_1	blaCTX-M-199_1
MG267386	China	chicken			Klebsiella pneumoniae	IS1380	mcr-7.1_1	blaCTX-M-55_1
KU932023	Finland	human			Escherichia coli			
KU932022	Finland	Human			Escherichia coli			
SRR3392791_18_1143	US				Salmonella enterica			

Supplemental Table S2 – MOB-suite analysis results

A - Full dataset:

MOB node	# per MOB	# with IS	% with IS	# with mcr	% with mcr
AI345	16	0	0.00	0	0
AI346	38	3	7.89	0	0
AI347	49	17	34.69	5	10.20
AI348	26	17	65.38	17	65.38

AI349	19	17	89.47	1	5.26
AI350	123	86	69.92	113	91.87

B – USA dataset

	Number	# with IS	% with IS	with mcr	Internal cluster percent
AI345	15	0	0	0	27
AI346	33	0	0	0	6
AI347	35	8	22.86	4	46
AI348	9	1	11.11	0	67
AI349	4	2	50	0	100
AI350	10	7	70	3	100

Supplemental Table S3: Core genes identified in USA collection

**Gene**	**Annotation**	**No. isolates**
virB1	TraB	271
pilM	conjugative transfer protein PilM	271
yheC	CaiF/GrlA family transcriptional regulator	271
yaeC	YaeC protein	271
yafA	YafA protein	271
traH	TraH	271
ygeA	YgeA	271
parA	plasmid partitioning protein ParA	271
nfeD	putative nodulation efficiency family protein	271
pilU	prepilin peptidase PilU	270
pilT	conjugative transfer putative membrane protein PilT	270
pilP	type IV pilus biogenesis protein PilP	270

yhaA	YhaA protein	270
pilO	IncI1 plasmid pilus assembly protein PilO	269
traI	TraI	268
traC	TraC	267
yddA	YddA protein	267
rcbA	Double-strand break reduction protein	267
pilL	conjugative transfer protein PilL	267
traK	TraK	267
traJ	TraJ	265
virB4	ATPase VirB4	264
pilN	conjugative transfer lipoprotein PilN	263
repA	replication initiation protein	262
ydfA	transcriptional regulator	261
group_225	homocysteine methyltransferase domain protein	261
yebA	YebA protein	260
traD	TraD	257
group_193	putative membrane protein	254

Supplemental Table S4: Metadata file - Linkage analysis AMOVA results. Populations compared are Group 1 and Group 2 from Figure 3.

	Among population:					Within populations:				Fixation indices:	
	Locus	SSD	d.f.	Va	% variation	SSD	d.f.	Vb	% variation	FST	P-value
DNA replication	1	2.41487	1	0.03922	8.43584	44.27381	104	0.42571	91.56416	0.08436	0
hypothetical	2	2.00171	1	0.03042	6.21694	47.7247	104	0.45889	93.78306	0.06217	0
hypothetical	3	2.22525	1	0.03533	7.53688	45.07664	104	0.43343	92.46312	0.07537	0

toxin-antitoxin system	7	2.75533	1	0.04672	10.80229	40.12202	104	0.38579	89.19771	0.10802	0
DNA -binding transcriptional regulator (prophage)	11	3.77111	1	0.08297	17.59672	31.46986	81	0.38852	82.40328	0.17597	0
rpoS stabilizer (hypothetical)	15	2.03647	1	0.04578	10.43655	32.21353	82	0.39285	89.56345	0.10437	0
lipoprotein (hypothetical)	20	3.31508	1	0.05829	13.98494	37.28869	104	0.35855	86.01506	0.13985	0
oxidoreductase, disulfide bond formation, phoP regulation	23	2.39167	1	0.04547	11.62779	32.48333	94	0.34557	88.37221	0.11628	0
spoVR family protein - sporolation	24	2.12314	1	0.0364	7.67999	41.12686	94	0.43752	92.32001	0.0768	0
conjugative transfer protein	26	2.37	1	0.07829	18.82372	16.88	50	0.3376	81.17628	0.18824	0
zinc transporter	27	3.25308	1	0.11304	26.20053	15.92	50	0.3184	73.79947	0.26201	0
nickase regulator	28	2.16608	1	0.03527	8.54871	39.23958	104	0.3773	91.45129	0.08549	0
nickase	29	1.36356	1	0.01743	3.5093	49.85342	104	0.47936	96.4907	0.03509	0
nickase	30	1.41323	1	0.02538	5.29747	39.93122	88	0.45376	94.70253	0.05297	0
nickase	31	2.81361	1	0.04902	13.0212	34.05432	104	0.32745	86.9788	0.13021	0
membrane transporter	35	2.49165	1	0.04044	8.40369	45.83854	104	0.44076	91.59631	0.08404	0

DNA topoisomerase	37	1.87302	1	0.02804	5.74692	47.36508	103	0.45986	94.25308	0.05747	0
hypothetical	38	2.79214	1	0.06831	16.13013	25.57273	72	0.35518	83.86987	0.1613	0
hypothetical	39	5.57097	1	0.13855	28.26602	27.0746	77	0.35162	71.73398	0.28266	0
hypothetical	40	2.45635	1	0.07993	18.29684	18.20402	51	0.35694	81.70316	0.18297	0
methionine sulfoxide reductase (membrane protein repair)	41	3.51216	1	0.06834	14.60895	38.34498	96	0.39943	85.39105	0.14609	0
murein DD-endopeptidase (cell wall expansion)	42	3.43082	1	0.06619	13.73632	39.90591	96	0.41569	86.26368	0.13736	0
shufflon-specific recombinase	43	3.67195	1	0.06702	14.57596	39.66786	101	0.39275	85.42404	0.14576	0
pilin - surface protein	44	3.68086	1	0.07921	21.77461	24.75734	87	0.28457	78.22539	0.21775	0
pilus modification	47	1.13968	1	0.01316	2.68727	49.07937	103	0.4765	97.31273	0.02687	0
pilus motility protein	48	1.85079	1	0.02768	5.72894	46.92063	103	0.45554	94.27106	0.05729	0
pilus motility protein	49	3.19683	1	0.05531	11.91306	42.12698	103	0.409	88.08694	0.11913	0
fimbriae regulatory protein	51	2.76825	1	0.0462	9.502	45.31746	103	0.43998	90.498	0.09502	0

pilus secretion	52	2.49048	1	0.04046	8.22437	46.5	103	0.45146	91.77563	0.08224	0
pilus assembly	53	2.15079	1	0.03386	7.08274	45.75397	103	0.44421	92.91726	0.07083	0
pilus assembly	54	2.6	1	0.04379	10.02608	40.47619	103	0.39297	89.97392	0.10026	0
pilus assembly	55	2.58413	1	0.04234	8.60061	46.34921	103	0.44999	91.39939	0.08601	0
D-serine permease (transporter)	56	2.26738	1	0.08608	17.73603	17.96667	45	0.39926	82.26397	0.17736	0
pilus assembly	57	2.44603	1	0.03949	7.97344	46.94444	103	0.45577	92.02656	0.07973	0
plasmid transfer regulator (conjugation)	58	3.17619	1	0.05431	11.00987	45.21429	103	0.43897	88.99013	0.1101	0
conjugative transfer relaxase	59	3.27349	1	0.05598	11.42232	45.15104	104	0.43414	88.57768	0.11422	0
conjugative transfer system protein	60	6.71629	1	0.12578	27.17404	35.05729	104	0.33709	72.82596	0.27174	0
conjugative transfer system protein	61	2.63931	1	0.04398	9.70948	42.53051	104	0.40895	90.29052	0.09709	0
conjugative transfer system protein	63	2.86603	1	0.04763	9.56363	46.84152	104	0.4504	90.43637	0.09564	0
conjugative transfer system protein	64	5.01485	1	0.09168	20.07718	37.95685	104	0.36497	79.92282	0.20077	0

conjugative transfer system protein	65	7.17081	1	0.13556	31.43253	30.75372	104	0.29571	68.56747	0.31433	0
pilus assembly	66	2.19786	1	0.03523	7.89512	42.74554	104	0.41101	92.10488	0.07895	0
racemase	67	3.71493	1	0.06501	13.46959	43.43601	104	0.41765	86.53041	0.1347	0
conjugative transfer system protein	69	2.83492	1	0.04745	9.66066	45.69841	103	0.44367	90.33934	0.09661	0
arginine transport	70	5.27597	1	0.09656	20.30925	39.40327	104	0.37888	79.69075	0.20309	0
ion channel	73	3.22347	1	0.05962	12.33597	40.67449	96	0.42369	87.66403	0.12336	0
ATPase	75	1.80944	1	0.02694	5.72791	46.10565	104	0.44332	94.27209	0.05728	0
conjugal transfer protein	76	1.89259	1	0.03849	8.08253	38.51852	88	0.43771	91.91747	0.08083	0
kinase	77	3.41936	1	0.06111	16.02709	33.29762	104	0.32017	83.97291	0.16027	0
hypothetical	78	2.82519	1	0.04822	11.27443	39.46726	104	0.37949	88.72557	0.11274	0
hypothetical	79	2.39844	1	0.06737	16.03914	24.33395	69	0.35267	83.96086	0.16039	0
membrane protein	80	3.67272	1	0.06594	16.7116	34.17634	104	0.32862	83.2884	0.16712	0
putative chaperone protein	81	2.58307	1	0.0427	9.28346	43.39807	104	0.41729	90.71654	0.09283	0
Rnase targeting protein	82	1.95888	1	0.02974	6.19441	46.84301	104	0.45041	93.80559	0.06194	0
hypothetical	87	3.02381	1	0.05246	12.13643	39.11905	103	0.3798	87.86357	0.12136	0
hypothetical	88	2.90092	1	0.06081	12.6244	33.67225	80	0.4209	87.3756	0.12624	0

putative transporter	89	1.94992	1	0.05954	15.03047	20.19524	60	0.33659	84.96953	0.1503	0
exodeoxyribonuclease V	101	1.77139	1	0.09042	19.47202	15.70588	42	0.37395	80.52798	0.19472	0
putative transcriptional regulator	109	3.43947	1	0.13377	29.27972	16.80127	52	0.3231	70.72028	0.2928	0
acyl-CoA esterase	110	3.45401	1	0.1348	30.0657	16.30525	52	0.31356	69.9343	0.30066	0
dihydroxyacetone kinase subunit	112	3.41915	1	0.13307	29.4208	16.59936	52	0.31922	70.5792	0.29421	0
hypothetical	117	2.14722	1	0.21291	45.53277	4.075	16	0.25469	54.46723	0.45533	0
conjugation regulator	8	2.56579	1	0.04372	11.14605	36.24554	104	0.34851	88.85395	0.11146	0.00098
toxin-antitoxin system	22	1.88813	1	0.03105	9.01436	32.59301	104	0.31339	90.98564	0.09014	0.00098
conjugative transfer system protein	62	0.81953	1	0.01341	8.76088	14.52009	104	0.13962	91.23912	0.08761	0.00098
prepilin leader peptidase/methylase	85	1.58081	1	0.02276	5.06368	44.37202	104	0.42665	94.93632	0.05064	0.00098
hypothetical	91	3.10898	1	0.10975	32.05313	13.02895	56	0.23266	67.94687	0.32053	0.00098
hypothetical	108	1.89583	1	0.29315	68.16609	1.91667	14	0.1369	31.83391	0.68166	0.00098
porin protein	113	2.58293	1	0.1493	37.46924	8.9697	36	0.24916	62.53076	0.37469	0.00098

diketo-D-gluconate reductase	10	1.82414	1	0.02826	6.74128	40.65699	104	0.39093	93.25872	0.06741	0.00196
hypothetical	25	1.21	1	0.02963	6.29818	22.04	50	0.4408	93.70182	0.06298	0.00196
pilin - surface protein	50	1.7	1	0.02591	6.16851	40.59524	103	0.39413	93.83149	0.06169	0.00196
hypothetical	105	1.46349	1	0.06999	15.73735	16.11429	43	0.37475	84.26265	0.15737	0.00293
site-specific recombinase	107	1.57192	1	0.07693	16.24709	13.48364	34	0.39658	83.75291	0.16247	0.00293
periplasmic endochitinase/lysozyme	84	1.55498	1	0.02291	5.51065	40.86012	104	0.39289	94.48935	0.05511	0.00391
hypothetical	104	1.74248	1	0.12194	32.51989	6.57895	26	0.25304	67.48011	0.3252	0.00391
exonuclease	16	1.57977	1	0.06484	16.98638	13.94196	44	0.31686	83.01362	0.16986	0.00489
hypothetical	90	1.40629	1	0.03913	9.45048	22.49694	60	0.37495	90.54952	0.0945	0.00489
pilus assembly	68	1.42787	1	0.0205	5.0171	40.36458	104	0.38812	94.9829	0.05017	0.00587
bacterioferritin-associated ferredoxin	83	1.57944	1	0.04131	10.19322	21.83991	60	0.364	89.80678	0.10193	0.0088
hypothetical	106	1.12424	1	0.08617	28.00396	4.20909	19	0.22153	71.99604	0.28004	0.01271
hypothetical	93	1.2578	1	0.0515	13.77617	11.28274	35	0.32236	86.22383	0.13776	0.01369
hypothetical	103	0.77778	1	0.0829	41.97802	1.83333	16	0.11458	58.02198	0.41978	0.01955
hypothetical	14	1.17481	1	0.02233	5.70849	30.61343	83	0.36884	94.29151	0.05708	0.02248
hypothetical	95	1.07285	1	0.04494	10.01194	20.19638	50	0.40393	89.98806	0.10012	0.02346
hypothetical	36	1.12041	1	0.01635	4.41472	35.03801	99	0.35392	95.58528	0.04415	0.02444
preprotein translocase	17	1.10038	1	0.02158	5.85192	29.85417	86	0.34714	94.14808	0.05852	0.0303

Diguanylate phosphodiesterase (transcriptional regulator)	12	0.9648	1	0.0157	3.79557	33.0352	83	0.39801	96.20443	0.03796	0.03715
hypothetical	118	1.04167	1	0.09773	20.82627	4.45833	12	0.37153	79.17373	0.20826	0.04301
antitoxin	72	0.90714	1	0.45	90	0.95	19	0.05	10	0.9	0.11144
hypothetical	13	0.60583	1	0.0077	2.29196	27.22947	83	0.32807	97.70804	0.02292	0.11339
toxin-antitoxin system	19	0.39	1	0.00831	4.17594	9.15	48	0.19062	95.82406	0.04176	0.15934
transcriptional regulator	100	0.55655	1	0.0126	3.17731	9.97917	26	0.38381	96.82269	0.03177	0.17889
transcriptional regulator	9	0.24809	1	0.00528	4.41481	5.82738	51	0.11426	95.58519	0.04415	0.22287
hypothetical	116	0.44444	1	0.03472	17.24138	2.66667	16	0.16667	82.75862	0.17241	0.23949
osmotically inducible protein	86	0.27242	1	0.00119	0.55555	22.07664	104	0.21228	99.44445	0.00556	0.24536
hypothetical	74	0.31837	1	0.00638	2.44807	8.89785	35	0.25422	97.55193	0.02448	0.25709
hypothetical	92	0.41224	1	0.00353	1.02273	16.05714	47	0.34164	98.97727	0.01023	0.26295
hypothetical	18	0.02484	1	0.00036	3.27456	0.96429	90	0.01071	96.72544	0.03275	0.3001
methionine metabolism	4	0.22133	1	0.00041	0.20129	20.88244	104	0.20079	99.79871	0.00201	0.32063
toxin-antitoxin system	21	0.04586	1	-0.00013	-0.24652	4.78571	93	0.05146	100.2465	-0.00247	0.39687
hypothetical	127	0.65	1	0.25	50	0.75	3	0.25	50	0.5	0.39883
multi-drug resistance	123	0.58333	1	0.16667	33.33333	0.66667	2	0.33333	66.66667	0.33333	0.50831

membrane protein											
pilus assembly	46	0.16855	1	-0.00321	-1.05249	26.46782	86	0.30777	101.0525	-0.01052	0.60704
hypothetical	45	0.16825	1	-0.00346	-1.09316	27.82051	87	0.31978	101.0932	-0.01093	0.62854
biofilm formation	96	0.0375	1	-0.07143	-71.4286	2.4	14	0.17143	171.4286	-0.71429	1
conjugation protein	125	0	1	-0.25	-100	1	2	0.5	200	-1	1
putative arabinose efflux transporter	169	0.08333	1	-0.16667	-100	0.66667	2	0.33333	200	-1	1
